# Theoretical basis for the use of non-invasive thermal measurements to assess the brain injury in newborns undergoing therapeutic hypothermia

**DOI:** 10.1038/s41598-020-79009-3

**Published:** 2020-12-17

**Authors:** Wojciech Walas, Dominika Bandoła, Ziemowit Ostrowski, Marek Rojczyk, Anna Mączko, Zenon Halaba, Andrzej J. Nowak

**Affiliations:** 1 Paediatric and Neonatal Intensive Care Unit, University Clinical Hospital, Opole, Poland; 2 Institute of Medical Sciences, University of Opole, Opole, Poland; 3 Department of Thermal Technology, Silesian University of Technology, Gliwice, Poland; 4 Department of Paediatrics, Institute of Medical Sciences, University of Opole, Opole, Poland

**Keywords:** Physiology, Health care

## Abstract

The aim of this paper is to propose a new non-invasive methodology to estimate thermogenesis in newborns with perinatal asphyxia (PA) undergoing therapeutic hypothermia (TH). Metabolic heat production (with respect to either a neonate’s body mass or its body surface) is calculated from the newborn’s heat balance, estimating all remaining terms of this heat balance utilising results of only non-invasive thermal measurements. The measurement devices work with standard equipment used for therapeutic hypothermia and are equipped with the Global System for Mobile Communications (GSM), which allows one to record and monitor the course of the therapy remotely (using an internet browser) without disturbing the medical personnel. This methodology allows one to estimate thermogenesis in newborns with perinatal asphyxia undergoing therapeutic hypothermia. It also offers information about instantaneous values of the rate of cooling together with values of remaining rates of heat transfer. It also shows the trend of any changes, which are recorded during treatment. Having information about all components of the heat balance one is able to facilitate comparison of results obtained for different patients, in whom these components may differ. The proposed method can be a new tool for measuring heat balance with the possibility of offering better predictions regarding short-term neurologic outcomes and tailored management in newborns treated by TH.

## Introduction

Despite recent advances in perinatal care, neonatal hypoxic-ischaemic encephalopathy (HIE) is one of the most common causes of severe neurological deficits in children, affecting approximately 15 per 10,000 live births^1^. According to^2^ perinatal asphyxia (PA) represents the second most common cause of neonatal (0–27 days) deaths in 2015. Therapeutic hypothermia (TH) is a method of treatment that has been proven effective in protecting the brain against the effects of ischaemia/hypoxia in neonates after PA^3–7^. The mechanism for such neurologic protection is thought to be multifactorial, including limitation of post-arrest endothelial dysfunction, decreased free radical release and blunting of the post-reperfusion inflammatory cascade^8^. In newborns only external hypothermia is practically used, i.e. the whole body cooling or selective head cooling. The assessment of the degree of brain damage and prognosis in newborns after PA is based on a complex analysis of different factors such as the Apgar score and neurological status during treatment, metabolic acidosis and other biochemical markers, electrophysiological tests-electroencephalography (EEG), also amplitude-integrated EEG, brainstem evoked potentials, and Near Infra-Red Spectroscopy. Neuroimaging, especially Magnetic Resonance Imaging plays a quite special role. Attention is drawn to the benefits of concurrently assessing the state of the brain using various methods^9–21^. However, all these tools have their limitations—none is fully sensitive and specific, some of them cannot be performed in the intensive care unit, others are expensive or they are at the stage of clinical suitability assessment. As a result, predicting the degree of injury and the possible long-term consequences of HIE remains challenging, especially during the first few days of illness. For these reasons, seeking additional ways to assess brain damage and prognostic factors is clinically relevant.

In both physiology and pathology, body temperature is the result of a heat balance, which includes endogenous heat production (thermogenesis) but also the exchange of heat with the local indoor environment. Body temperature regulation is effected primarily through dedicated pathways in the brain^22–24^. It is also a result of heat exchange between newborn treated in intensive care unit and the indoor environment which is of a particularly complex nature, mainly due to the use of incubators and radiant warmers^25^.

In several studies estimates of heat production were identified by changes of temperature of the water abstracted from cooling devices. Some theoretical limits on the brain cooling by external head cooling devices have been discussed by Sukstanskii and Yablonskiy^26^. It has been shown that heat production estimated in this way correlates with HIE severity in adult patients after cardiac arrest^27,28^. Similar results were obtained in the study of neonates with PA^29^. It should, however, be underlined that considering only the water-cooling process, some other heat transfer mechanisms have been ignored.

Therefore, there is no doubt that HIE is associated with impairment in thermoregulation and spontaneous hypothermia. Based on these premises, we suppose that in newborns with PA, treated with TH, assessment of endogenous heat production could be an additional useful prognostic factor. Thus, there is an urgent need to increase and strengthen the knowledge about heat transfer processes that occur within the neonate’s body but also within the local indoor environment during the therapy.

The aim of this paper is to propose a new non-invasive methodology to estimate thermogenesis of newborns with HIE undergoing TH. Heat production (with respect to either a neonate’s body mass or its body surface) is calculated from the neonate’s heat balance, estimating all remaining terms of this balance, utilising results of again only non-invasive thermal measurements. A small number of results obtained in measurements (carried out by the University Clinical Hospital in Opole, Poland) are used in this work. The role of these measurements is twofold: they serve as exemplary input data and as a preliminary test of the method performance. The whole methodology, as well as all the measurements carried out, have obtained acceptance of the bioethics committee of the Opole Medical Chamber (Resolutions nos 271/2018 and 272/2018).

The main idea and theoretical considerations of the proposed methodology, together with the important steps of TH and required measurements are briefly discussed in the next section. Then, some exemplary results are demonstrated. Finally, some concluding remarks are presented.

## Methodology

### Heat balance for a neonate undergoing TH

It is assumed that for a neonate undergoing TH the rate of external mechanical work accomplished can be neglected. Hence, the heat balance for the neonate can be written as^30,31^:

1 $$Q_{m} = \frac{{{\text{d}}U}}{{{\text{d}}\tau }} + Q_{skin} + Q_{resp}$$

where *Q*_*m*_ represents the rate of metabolic heat production, *Q*_*skin*_ is the rate of heat dissipated through the skin (to the local indoor environment) while *Q*_*resp*_ stands for the rate of heat exchanged due to respiration. These three rates are all expressed in watts (W). The time derivative on the right-hand side of the equation accounts for changes of internal energy *U* (depending on temperature) with respect to time and can be split into two terms related to the skin compartment and to the core compartment:

2 $$\frac{{{\text{d}}U}}{{{\text{d}}\tau }} = \left. {\frac{{{\text{d}}U}}{{{\text{d}}\tau }}} \right|_{skin} + \left. {\frac{{{\text{d}}U}}{{{\text{d}}\tau }}} \right|_{core} = \left( {1 - \alpha_{skin} } \right)\,m\,c_{b} \left. {\frac{{{\text{d}}T}}{{{\text{d}}\tau }}} \right|_{skin} + \left. {\alpha_{skin} \,m\,c_{b} \frac{{{\text{d}}T}}{{{\text{d}}\tau }}} \right|_{core}$$

Symbol *m* represents the neonate’s body mass, *c*_*b*_ is the specific heat of the tissue (typically *c*_*b*_ = 3490 J kg^−1^ K^−1^) while coefficient *α*_*skin*_ accounts for the rate of blood flowing to the skin^30,31^. Both temporal derivatives of temperature with respect to time *τ* can easily be calculated numerically (e.g. as the central difference quotient was used over a 2-min time step) taking into account core and skin temperatures recorded during therapy by the cooling device.

It is important to notice that for a neonate undergoing TH, the heat rate transmitted to the cooling medium pumped by cooling device *Q*_*cooling*_ (no matter whether this treatment is carried out as a whole body cooling or selective head cooling) is a dominant part of the rate of heat dissipated through the neonate’s skin *Q*_*skin*_. Hence, this term is written explicitly, next to a classical rate of heat dissipated by conduction *Q*_*cond*_, convection *Q*_*conv*_, thermal radiation *Q*_*rad*_ and evaporative heat loss from skin *Q*_*skin-evap*_^30,31^, i.e.

3 $$Q_{skin} = Q_{cooling} + Q_{cond} + Q_{conv} + Q_{rad} + Q_{skin{\text{-}}evap}$$

The heat rate transmitted to the cooling medium *Q*_*cooling*_ depends on the temperature of the water flowing into the cooling element (i.e. either cooling mattress, cooling pad or cooling cap) and flowing out of it, but also on the water flow rate. All these three quantities can easily be estimated utilising results of non-invasive measurements described in the next subsection.

Bearing in mind that the rate of heat dissipated by conduction *Q*_*cond*_ accounts for heat transfer other than through the cooling system it is clear that this term is generally quite small and might be neglected.

The rate of heat exchanged between the neonate’s skin and surrounding air by convection *Q*_*conv*_ depends on this temperature difference *∆T*_*conv*_, heat transfer coefficient *h*_*conv*_ (in W m^−2^ K^−1^) and body surface area *A*_*conv*_ (in m^2^).

4 $$Q_{conv} = A_{conv} \,h_{conv} \,\left( {T_{skin} - T_{sur} } \right) = A_{conv} \,h_{conv} \,\Delta T_{conv}$$

The total body surface area (BSA) can easily be obtained from many formulae, e.g. Du Bois formuladu bois^32^. However, in determining *Q*_*conv*_ one can be more precise and follow the idea of sectioning of the whole body surface commonly used for estimation the size of a burn^33,34^. Each distinguished area/section has its own temperature. The heat transfer coefficient has already been measured for newborns treated within an open incubator^35^.

Neonates exchange heat also by radiation. The rate of heat exchanged by radiation *Q*_*rad*_ depends on the difference of the fourth power of skin temperature *T*_*skin*_ and the fourth power of temperature, representing the whole local indoor environment *T*_*rad*_ (so-called radiant temperature). This latter temperature can be measured using a globe thermometer and this is described in the next subsection. In the formula defining *Q*_*rad*_ skin emissivity *ε*_*skin*_ and body surface active area *A*_*rad*_ (estimated similarly to the case of convection) is also involved, i.e.

5 $$Q_{rad} = A_{rad} \,\sigma \,\varepsilon_{skin} \,\left( {T_{skin}^{4} - T_{rad}^{4} } \right)$$

The last term in Eq. (3), i.e. *Q*_*skin-evap*_, depends on the body surface active area *A*_*evap*_, the evaporative heat transfer coefficient *h*_*evap*_ (in W m^−2^ Pa^−1^), the skin wittedness *w* (dimensionless) and on the difference of water vapour pressure *∆p* (in Pa) in the air very close to the neonate’s skin and in the room environment.

6 $$Q_{skin{\text{-}}evap} = A_{evap} \,h_{evap} \,w\,\Delta p$$

These water vapour pressures are directly linked to skin and air temperatures. Details can be found in^30,31^.

Although, the rate of heat exchange due to respiration *Q*_*resp*_ can be split into two terms: the rate of convective heat transfer and the rate of evaporative heat transfer it is important to realise that the total rate of heat transfer due to respiration *Q*_*resp*_ is proportional to the pulmonary ventilation rate *m*_*resp*_ (in kg s^−1^) and to the difference of enthalpy of exhaled and inhaled breathing mixture *∆h*_*e*_ (in J kg^−1^, dry breathing air), i.e.

7 $$Q_{resp} = m_{resp} \,\left( {h_{e - ex} - h_{e - in} } \right) = m_{resp} \,\Delta h_{e}$$

In the case of apneic newborns undergoing TH, the pulmonary ventilation rate *m*_*resp*_ (being a product of the tidal volume and the respiratory rate) is fully controlled by the respirator and is known exactly. For other types of respiratory support, the pulmonary ventilation rate is not known exactly but even in this case *m*_*resp*_ can still be estimated by relating it to the metabolic rate^3^

8 $$m_{resp} \, = K_{res} \,Q_{m}$$

where *K*_*res*_ stands for the proportionality coefficient.

It is also worth noting that during the moisturising process, independently on various types of mechanical ventilation and/or non-invasive respiratory support, the breathing mixture is humidified and heated to about 37 °C. This means in practice that the temperature of the inspired air is above the neonate’s core temperature and the enthalpy difference is negative (*∆h* < 0 and *Q*_*resp*_ is in fact subtracted from the summation of other terms). As a consequence, the need for heated and humidified air in the ventilator circuit as necessary to ventilation though at odds with the competing interest of hypothermia.

It is also important to say that the total rate of heat transfer due to respiration *Q*_*resp*_ determined based on parameters of exhaled and inhaled breathing mixture is in practice considerably smaller compared to other terms of heat balance.

For prognostic purposes, the rate of metabolic heat production *Q*_*m*_ estimated in this way needs to be related either to the neonate’s body mass or its body surface.

### Course of the therapeutic hypothermia

Each specific TH has its own peculiarities. Nevertheless, there are several actions/steps which are very similar and important from a heat transfer point of view. These important steps for TH carried out using Olympic Cool-Cap (Olympic Medical Corp., USA) and/or Arctic Sun 5000 (Medivance, Inc., USA), just as examples of two representatives of selective and whole body TH, are briefly described below.

The essence of TH is to maintain the newborn's core temperature at a reduced level, which in the case of selective head hypothermia is 34–35 °C, and 33–34 °C in the case of whole body hypothermia. This can be achieved in different ways, depending on the type of cooling device. In both cases, the newborn is placed in an open incubator, and in both cases, the treatment lasts 72 h.

Olympic Cool-Cap is a device used to perform TH by selective head cooling, in which medical staff manually regulate the patient's temperature. The cooling element is a water-cooled cap that is put on the newborn's head. The patient's temperature is measured in three places: the rectal sensor measures the core temperature, and two sensors measuring the surface temperature are located on the abdomen and scalp, respectively. Patient data should be defined and entered into the device memory before cooling begins. Based on the weight of the newborn, the device pre-sets the temperature of the water flowing through the cap. While conducting TH, medical staff controls the patient's core temperature and maintains it within 34–35°C manually by changing the incubator radiant heater settings. In special situations, when despite the radiant heater adjustment settings it is not possible to maintain the core temperature in the desired therapeutic range, it is possible to manually change the mean temperature of the water flowing through the cap. After 72 h, TH ends, and the patient is rewarmed to physiological temperature. Rewarming is carried out manually and should be slow, not faster than 0.5 °C h^−1^, so it usually lasts at least 4 h. The cooling cap is removed from the newborn's head and the radiant warmer is adjusted manually to achieve the desired rate of increase of the newborn's core temperature. The remaining two surface temperature sensors (i.e. on the abdomen and on the scalp) continuously record these measurements during TH, although these two measurements are usually not utilised by neonatologists for treatment.

Arctic Sun 5000 is a device used to perform TH by whole body cooling, in which the patient's temperature is controlled automatically. The cooling element is a water-cooled mattress on which the newborn is laid or the pad, which the patient is wrapped in. The patient's core temperature is measured with a rectal sensor and displayed on the device screen. In addition, the device displays the temperature and velocity of the water flowing into the mattress/pad, although these measurements are usually not utilised by neonatologists for treatment. The physician sets the desired newborn's core temperature (33–34°C), and the device maintains it automatically, changing the temperature of the cooling water depending on the current core temperature measured by the rectal sensor. As with selective head hypothermia, after 72 h, TH ends, and the patient is rewarmed to physiological temperature. Heating occurs a bit slower, usually 0.25–0.5 °C h^−1^, so it usually lasts about 6–12 h. The newborn baby stays on the mattress or wrapped with pads. The physician sets the desired rewarming rate and the device does it automatically, based on the measured core temperature, by regulating the temperature of the water flowing through the mattress/pads. During TH and the rewarming process, two surface temperature sensors (i.e. abdomen and scalp) continuously record the result of these measurements. Additionally, during TH and the rewarming process, medical staff can still observe the temperature and velocity of the water flowing into the mattress/pad.

### Non-invasive measurements

**Figure 1:**
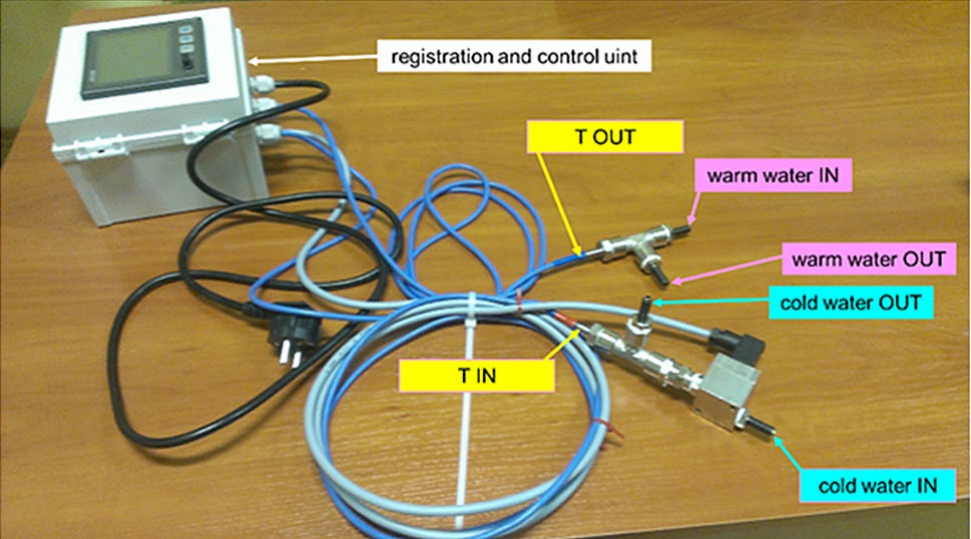
A water flow meter equipped with temperature sensors to monitor fluctuations of heat flux during therapy remotely.

Discussion of non-intensive measurements starts with the most important (i.e. dominant) term *Q*_*cooling*_ in Eq. (3). To be able to determine this quantity with appropriate accuracy a special water flow meter equipped with temperature sensors is recommended and shown in Fig. 1.

**Figure 2:**
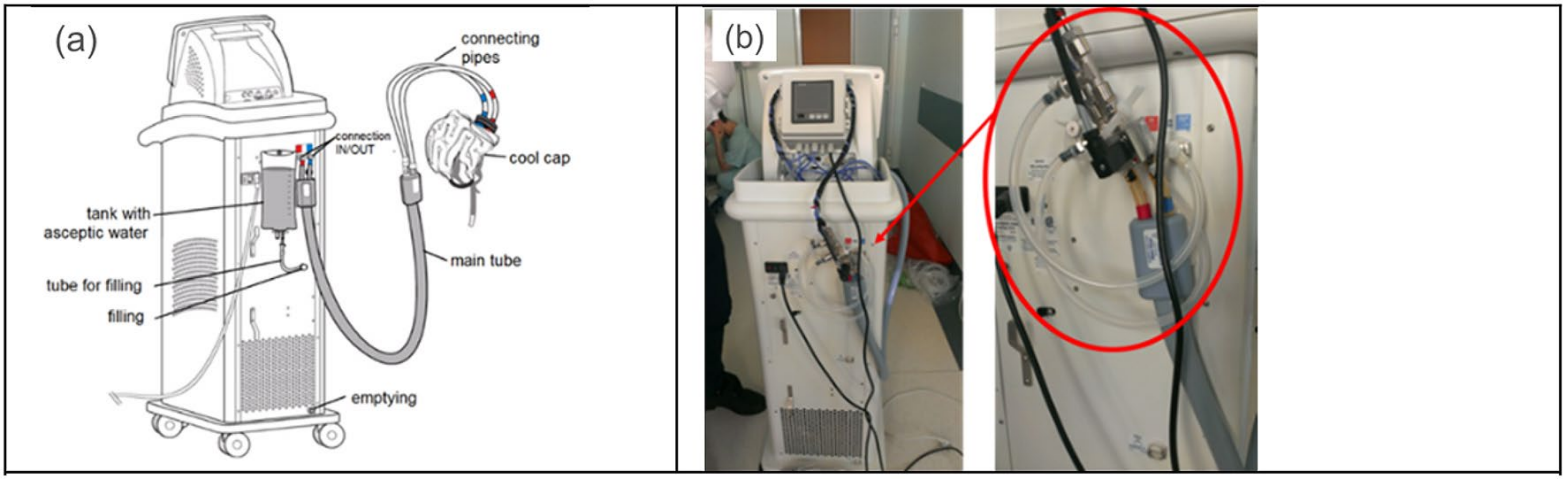
(**a**) Selective head cooling device; (**b**) device equipped with the water flow meter and temperature sensors.

Figure 2a,b demonstrate where and how this water flow meter equipped with temperature sensors has been connected to one of IN/OUT ports of the selective head cooling device.

The meter is equipped with a Global System for Mobile Communications (GSM) and allows one to monitor fluctuations of heat flux *Q*_*cooling*_ during therapy even remotely using an internet browser. In this way all calculations and analyses carried out during therapy can be made without any disturbance to medical personnel. It should be stressed that all recorded data are transferred via an encrypted connection and are secured by user account and password. Hence, from the therapeutic point of view, there are no differences and medical staff can operate in a standard way.

**Figure 3:**
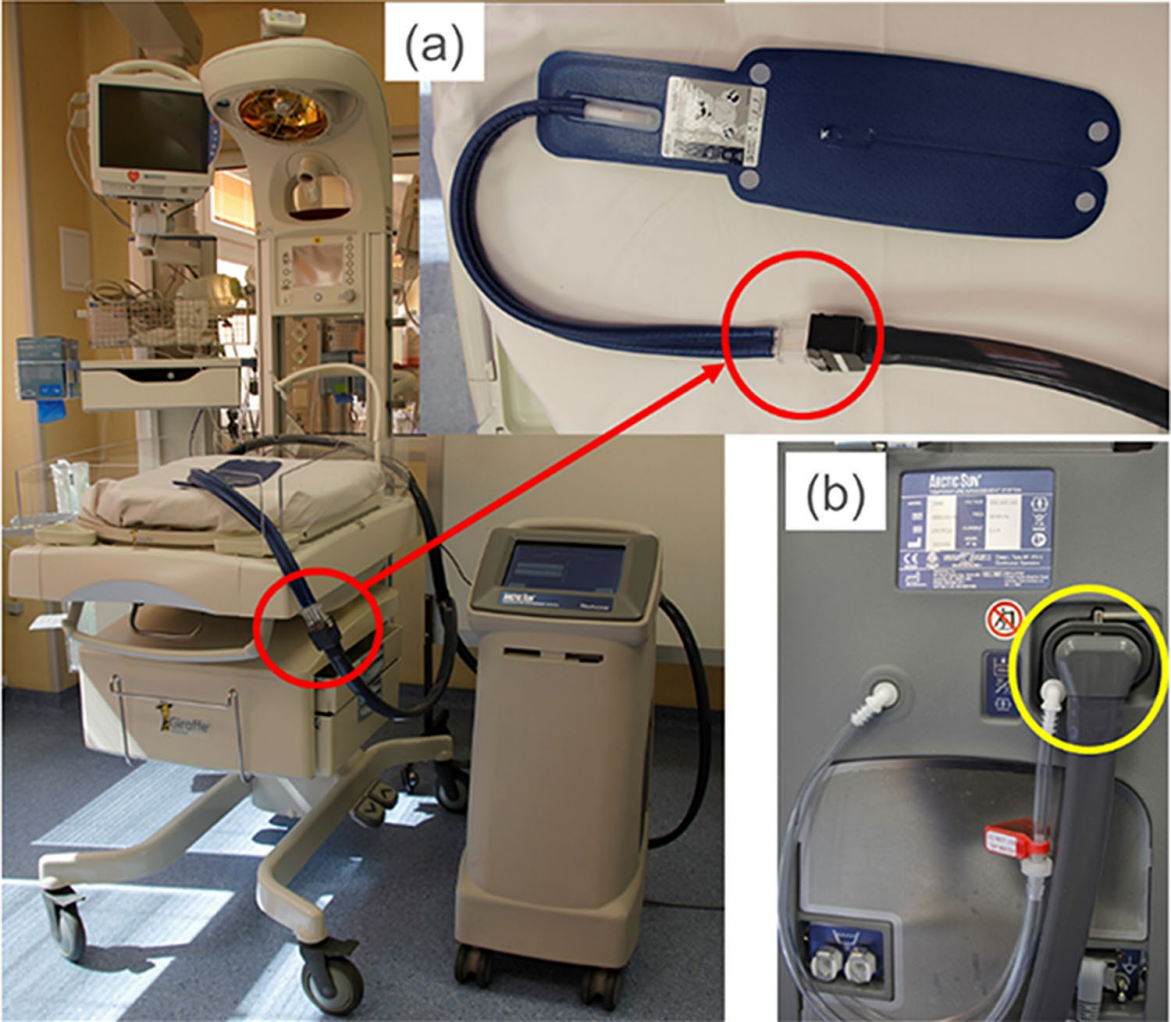
Whole body cooling device—(**a**) the main tube with connections; (**b**) port on the back of control unit.

For whole body cooling, sensors can be connected in a similar way, i.e. between control unit and cooling element. This is schematically shown in Fig. 3, either at position (a) of the main tube or at port (b) on the back of control unit.

**Figure 4:**
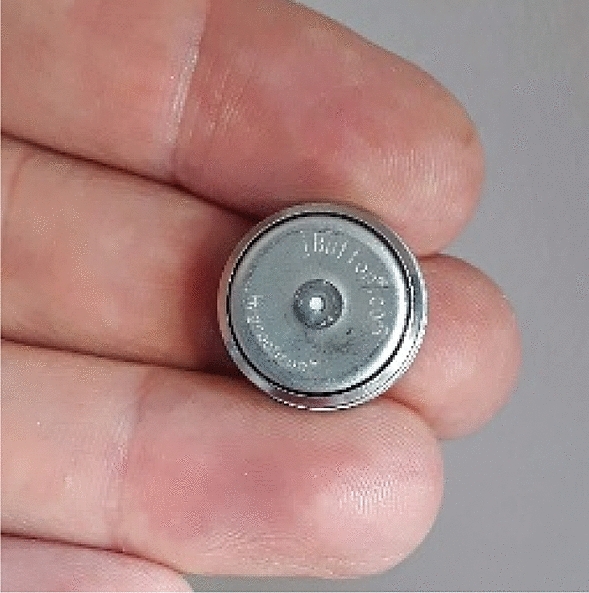
iButton wireless temperature and humidity recorder.

Temperature and relative humidity of the air in the medical room can be measured continuously by so-called iButton (Maxim Integrated Inc., USA) humidity and temperature recorders, shown in Fig. 4. Knowing these parameters and combining them with temperatures of the skin (abdomen and forehead) the rate of heat exchanged between the neonate’s skin and surrounding air by convection *Q*_*conv*_ as well as due to evaporation of water from the skin *Q*_*skin-evap*_ can be estimated.

**Figure 5:**
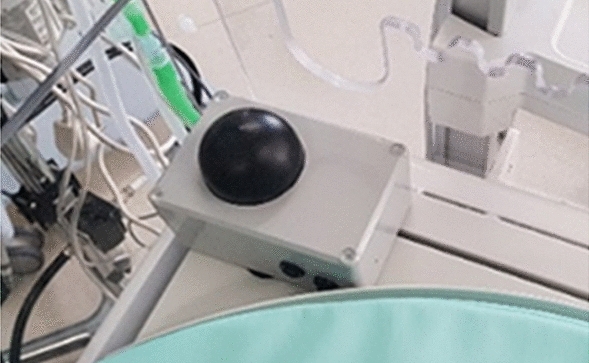
Globe thermometer (radiant temperature sensor) placed in the infant care bed.

Apparent temperature of all elements irradiating the patient in the medical room can be measured and collected using the globe thermometer. Its modified (custom-made) version is shown in Fig. 5.

Again, knowing the radiant temperature and combining it with temperatures of the skin (above abdomen and forehead) the rate of heat exchanged by radiation *Q*_*rad*_ can be estimated.

Additional data, related to the inhaled breathing air come from the respirator working conditions. It is commonly assumed that exhaled air reaches a saturation state defined by the core temperature and then the estimation of the difference of enthalpy *∆h* can be assumed based on humid air properties tables.

### The ethical statements

The study was approved by the bioethics committee of the Opole Medical Chamber (Resolutions nos 271/2018 and 272/2018). All methods were performed in accordance with the relevant guidelines and regulations.

All guardians were given an information form and gave their approval for the study, and written informed consent was obtained from the parents or legal guardians of all participating patients.

Measurements have been carried out courtesy of University Clinical Hospital in Opole, Poland. Data are available on request from the authors.

## Representative results

To demonstrate the main steps of the proposed methodology, all the non-invasive thermal measurements and corresponding calculations described in “Methodology” have been carried out for one neonate undergoing TH and will be discussed here.

A female infant was born at 40 weeks of gestation. The body mass was 3.130 kg while the length was 54 cm. After relevant medical examination patient has been qualified for selective head TH (using the Olympic Cool-Cap device). Treatment started on the birth day and lasted for 72 h.

**Figure 6:**
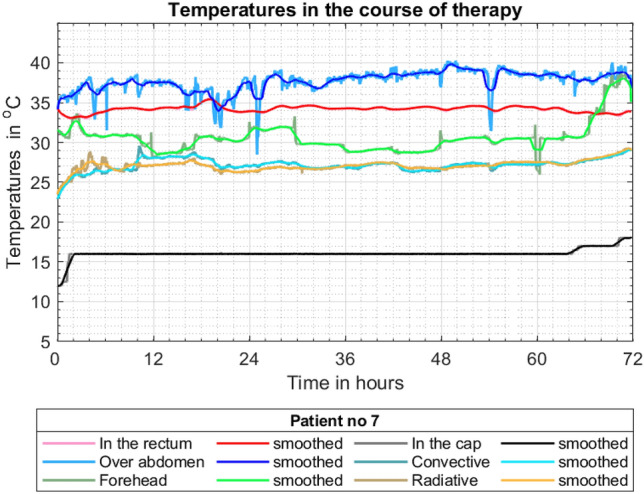
Selected temperatures in the course of therapy.

The following standard temperatures characterising treatment have been recorded: the temperature in the rectum, the temperature of the skin over the abdomen and temperature of the skin on the forehead. Radiant and convective temperatures described in “Heat balance for a neonate undergoing TH”, have also been recorded using a custom-made globe thermometer unit. All these temperatures, together with the mean temperature of the water flowing through the cooling cap, are shown in Fig. 6. Darker colours indicate fluctuations of the particular temperatures while brighter colours correspond to curves obtained after smoothing process. Smoothing was done using a standard MATLAB (The MathWorks, Inc., USA) moving average filter (built-in function ‘smooth’) with span equals to 0.02 for approx. 4350 data points.

It should be noted that the core temperature, represented by the red curve, generally falls within the required range, i.e. 34–35°C and the mean temperature of the cooling water remains constant. All corrections/modifications of the core temperature were achieved by changes of the radiant warmer power visible in Fig. 6 as changes of the radiative and convective temperatures. Only at the end of the treatment, (hour 64+) was the alteration of the water mean temperature needed.

It should also be noted that the temperature of the skin on the forehead (indicated by greenish lines) generally oscillates around 30 °C. It has regular (approx. every 12 h) temporary drops. This is the result of a routine procedure of removing the cooling cap and examining the state of head skin every 12 h. Other sudden decreases of temperature, visible also on blue lines, might be results of the application of some fluid boluses at room temperature.

**Figure 7:**
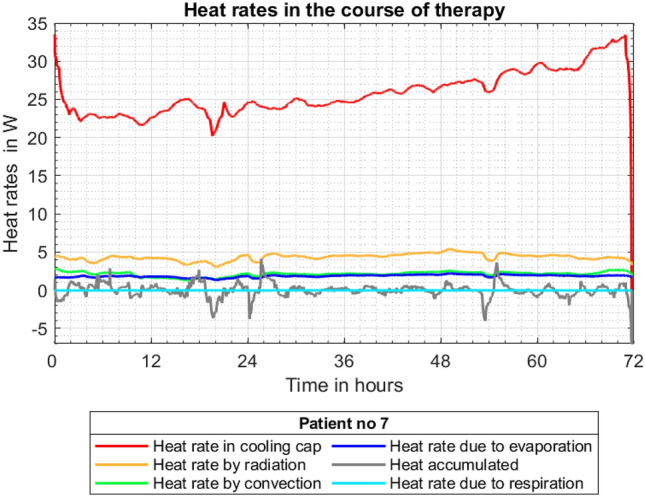
Heat rates in the course of therapy.

Based on temperature histories presented in Fig. 6 all heat rates discussed in “Heat balance for a neonate undergoing TH” have been calculated and are shown in Fig. 7. This graph demonstrates the contributions of particular heat rates to global metabolic heat production.

As expected the dominant effect is the heat rate retrieved by the cooling water *Q*_*cooling*_, indicated by the red line. The next most dominant, but much less so, is the heat rate transferred by radiation *Q*_*rad*_. The heat rates transferred by convection *Q*_*conv*_ and by the water evaporation through the neonate’s skin *Q*_*skin-evap*_ are on a similar level. The accumulation term oscillates around 0 W since heat transfer processes (except the heat flux to the cooling water) quickly reach an almost steady state. As already mentioned the heat rate due to respiration *Q*_*resp*_ is negative but its contribution is very small.

**Figure 8:**
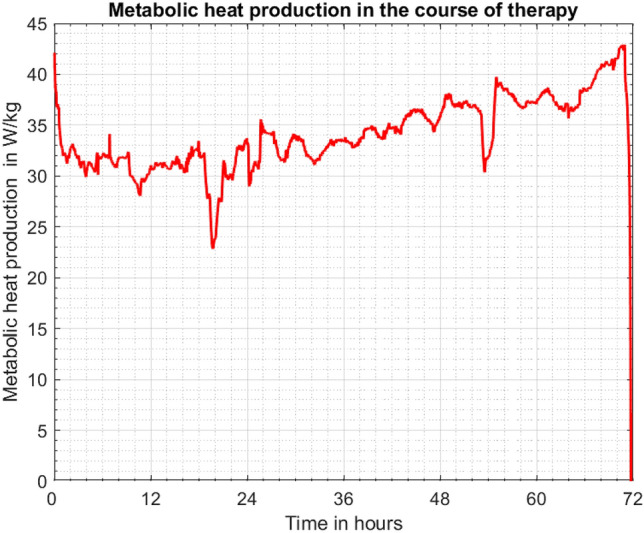
Metabolic heat production related to neonate’s body mass.

The global metabolic heat production is certainly the summation of all lines plotted in Fig. 7. Relating this quantity to the neonate’s body mass one can obtain the graph shown in Fig. 8.

## Discussion and concluding remarks

The thermoregulation centre is located in the brain, in the hypothalamus^22–24^. It has been shown that the rarely diagnosed Shapiro syndrome (spontaneous periodic hypothermia) is associated with hypothalamic dysfunction^36–40^. It has already been shown that in patients with brain injury (both traumatic and non-traumatic), thermoregulation disorders occur^41–43^. It has been proven that in adult patients with traumatic brain injury and after cardiac arrest (CA), thermoregulation disorder expressed as spontaneous hypothermia is associated with a worse prognosis: lower temperature before the start of TH, shorter time needed to reach the TH target temperatures and longer passive rewarming time are all associated with a worse outcome of the therapy^44–53^. This indicates that reduced heat production after CA arrest worsens prognosis.

Murnin^27^ estimated the endogenous heat production in adult patients treated with TH after CA. Patients' heat generation has been linked to so-called “Heat Index” (HI), calculated as a scaling factor equal to 100 over an average temperature of cooling medium (i.e. water) pumped by cooling device. That ratio was calculated for a 2-h period when the patient’s temperature was stable and equal to the target cooling medium temperature of TH treatment. Murnin et al.^27^ claim that HI calculated using Eq. (8) corresponds to the patient’s metabolic heat generation rate as the higher HI corresponds to the necessity of using a colder medium, i.e. increased patient-coolant temperature difference, to keep patient in the target TH treatment temperature. Although this last conclusion looks natural and acceptable, the Heat Index itself as a direct quantification of the metabolic heat generation rate might not be enough. The heat transfer rate is generally driven by the themperature difference but depend also on body mass. In any case, a positive correlation between neurological injuries and lower metabolic heat generation rates seems to be worth studying and it is not surprising that the HI was proposed by Murnin and co-workers as a prognostic tool for neurologically intact survival of patients after CA.

These observations were confirmed by Uber et al.^28^, who also used HI. They demonstrated that increased energy required by a cooling device to cool a patient after CA to target temperature is associated with improved outcomes at hospital discharge. The phenomenon of spontaneous hypothermia after PA has been known for a long time, but although it is known among medical staff that a deeper spontaneous drop in temperature is associated with more severe brain damage, there are few publications on this subject^54–61^.

Additional information on thermoregulation disorders in neonates after PA comes from TH studies. It was found that infants after PA with more severe encephalopathy had a lower temperature prior to the induction of active cooling, and neonates who were not qualified for TH had a tendency to spontaneous hypothermiaroberts^62–64^. It has also been noted that passive cooling of newborns after PA, which is sometimes used during transport, is often sufficient to reach the desired temperature^65–67^.

Mietzsch^29^ also noted that in newborns with PA treated by TH, temperature of the active cooling medium correlates with severity of brain injury diagnosed with Magnetic Resonance Imaging (MRI). Namely, the temperature of the cooling medium required to keep patients’ at target temperature of 33.5 °C was found and proposed to be a prognostic factor for neurological injuries in neonates with HIE. Taking into account that patients with improved outcomes required at any stage of TH the cooling medium temperature lower than 32 °C, the authors concluded that this might be a factor indicating neurological intact survival.

The methodology that has been developed and proposed in the present paper generally refers to the works of Murnin^27^ and Uber^28^ (for adults) and Mietzsh^29^ (for newborns). It should be stressed, however, that the proposed approach takes into account many more aspects and parameters/factors affecting the heat transfer processes occurring during TH. It should be expected that the complete heat balance for the neonate is a better-suited method to assess endogenous heat production than only information about the temperature of one medium and consequently about one term of this balance, even if this term is principal. It should also be noted that the proposed approach, supported with non-invasive measurements, offers not only an instantaneous value of the rate of cooling heat but also trends of changes which are recorded. Taking into account several components of the heat balance should facilitate the comparison of results obtained in different patients, in whom these components may differ. It is also very important that the measurement devices are equipped with a GSM system which allows one to monitor the course of the therapy remotely (using internet browser) without disturbing medical personnel^68–70^.

Application of the heat balance for newborn infants nursed under radiant warmers to analyse their physiological strain has also been discussed by Molgat-Seon^41^. However, these patients were not undergoing any therapeutic hypothermia and the heat rates estimated by the authors cannot be directly transferred nor compared to patients considered in this paper. Moreover, in the cited work the rate of metabolic heat was estimated using equations of indirect calorimetry, while in the approach proposed here the total metabolic heat is a result of the summation of components of Eq. (1), namely Eqs. (2)–(7), including measurement of a rate of heat that is removed by a cooling device (a cap or a mattress).

The research presented in this paper is to be continued. To confirm the clinical usefulness of the proposed method, it is necessary to perform investigations that correlate the results of measurements with the clinical condition of patients and with other tools assessing the degree of brain damage, as well as with the development of newborns. The proposed method can be a new tool for measuring heat balance with the possibility of offering better predictions regarding short-term neurologic outcomes and tailored management in newborns treated by TH.

## Data Availability

All calculations and presentations of results have been prepared using Matlab.

## List of symbols

*A*: Surface area
*c*: Specific heat
*h*: Heat transfer coefficient
*h*_*e*_: Enthalpy of breathing mixture
*m*: Mass
*m*_*resp*_: Pulmonary ventilation rate
*Q*: Rate of heat
*T*: Temperature
*U*: Rate of internal energy
*α*_*skin*_: Rate of blood flowing to the skin
*ε*_*skin*_: Skin emissivity
*σ*: Radiation constant
*τ*: Time
